# Why Does the Face Predict the Brain? Neural Crest Induction, Craniofacial Morphogenesis, and Neural Circuit Development

**DOI:** 10.3389/fphys.2020.610970

**Published:** 2020-12-11

**Authors:** Anthony-Samuel LaMantia

**Affiliations:** ^1^ Laboratory of Developmental Disorders and Genetics and Center for Neurobiology Research, Fralin Biomedical Research Institute, Department of Pediatrics, Virginia Tech-Carilion School of Medicine, Virginia Tech, Roanoke, VA, United States; ^2^ Department of Biological Sciences, Virginia Tech, Blacksburg, VA, United States

**Keywords:** neural crest, placodes, olfactory, sensory pathways, inductive signaling, 22q11 deletion syndrome

## Abstract

Mesenchephalic and rhombencephalic neural crest cells generate the craniofacial skeleton, special sensory organs, and subsets of cranial sensory receptor neurons. They do so while preserving the anterior-posterior (A-P) identity of their neural tube origins. This organizational principle is paralleled by central nervous system circuits that receive and process information from facial structures whose A-P identity is in register with that in the brain. Prior to morphogenesis of the face and its circuits, however, neural crest cells act as “inductive ambassadors” from distinct regions of the neural tube to induce differentiation of target craniofacial domains and establish an initial interface between the brain and face. At every site of bilateral, non-axial secondary induction, neural crest constitutes all or some of the mesenchymal compartment for non-axial mesenchymal/epithelial (M/E) interactions. Thus, for epithelial domains in the craniofacial primordia, aortic arches, limbs, the spinal cord, and the forebrain (Fb), neural crest-derived mesenchymal cells establish local sources of inductive signaling molecules that drive morphogenesis and cellular differentiation. This common mechanism for building brains, faces, limbs, and hearts, A-P axis specified, neural crest-mediated M/E induction, coordinates differentiation of distal structures, peripheral neurons that provide their sensory or autonomic innervation in some cases, and central neural circuits that regulate their behavioral functions. The essential role of this neural crest-mediated mechanism identifies it as a prime target for pathogenesis in a broad range of neurodevelopmental disorders. Thus, the face and the brain “predict” one another, and this mutual developmental relationship provides a key target for disruption by developmental pathology.

## Introduction

Nearly 60 years ago, Demyer et al. (1964) published a description of a series of cases with varying degrees of craniofacial malformations: from near cyclopia in still born fetuses to two patients, described in detail, with mild, but detectable, craniofacial anomalies. Based upon limited clinical observations, they argued that the degree of craniofacial malformation in these individuals correlated with brain dysmorphology and dysfunction. This apparent relationship led them to conclude, in a memorable – if not fully appreciated – title that “The Face Predicts the Brain.” The subtitle of their paper was prescient: “Diagnostic Significance of Median Facial Anomalies for Holoprosencephaly (Arhinencephaly).” The mechanistic significance of this relationship, including the consequences for the olfactory periphery (the nose) and its forebrain (Fb) targets [the olfactory bulbs (OBs) and other basal Fb nuclei], as well as additional peripheral sensory, brainstem, cerebral cortical, or basal Fb structures and circuits, however, was unclear at the time. Subsequent studies from the late 1980s onward give cell biological and molecular definition to the 1964 provocative idea of DeMyer et al. The face does indeed predict the brain. The central role of the neural crest in this predictive relationship is the subject of this review.

**Figure 1:**
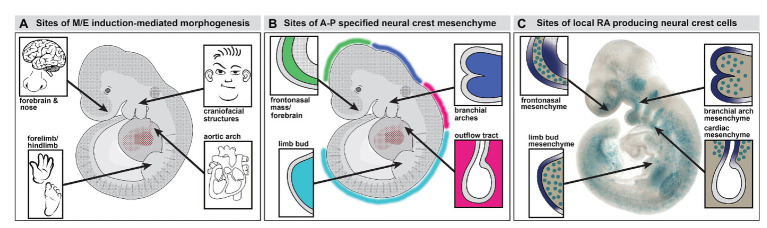
Neural crest mediated mesenchymal/epithelial (M/E) induction prefigures nasal/forebrain (Fb), craniofacial, heart, and limb morphogenesis. **(A)** A summary of the sites of non-axial M/E induction and their morphogenetic endpoints. The arrows point to the embryonic regions illustrated in panel B. **(B)** A summary of the relationship between anterior-posterior (A-P) regionally specified neural crest and the sites of M/E induction that establish the nose and Fb, the face, the heart, and the limbs. At each site, a primarily neural crest-derived population of A-P specified mesenchymal cells is opposed to the adjacent surface ectoderm, which is also axially specified. **(C)** Subsets of neural crest-derived mesenchymal cells, labeled with a knock-in reporter transgene (βgeo6; LaMantia et al., 2000; Bhasin et al., 2003) at each of the sites of M/E induction produce the morphogenetic signaling molecule retinoic acid (RA). These cells drive locally patterned expression of several target genes in placodal domains (purple shading) immediately adjacent to the RA-producing mesenchymal cells.

If the face predicts the brain, it is essential to define the nature of the prediction. This relationship reflects the central role of interactions between the craniofacial primordia, cranial placodes, and the rhombencephalic and mesencephalic neural crests, which provides a “mirror” representation of the axial organization of the neural tube to distal sites of secondary induction and differentiation: the facial skeleton and cartilage, key sensory structures, like the nose, eyes, ears, and cranial ganglia, and their targets in the central nervous system. Parallel neural crest-mediated interactions influence the aortic arches that become the great vessels of the heart, and this mechanism also influences limb bud patterning and differentiation (Figure 1A). This “mirror” representation of the early developing brain casts its reflection by localizing cardinal signaling molecules: retinoic acid (RA; Richman, 1992; Morriss-Kay, 1993; Rawson and LaMantia, 2006; Williams and Bohnsack, 2019), Fgfs (Tucker et al., 1999; Nie et al., 2006b; Szabo-Rogers et al., 2008; Stanier and Pauws, 2012), Shh (Hardcastle et al., 1999; Nasrallah and Golden, 2001; Smith et al., 2014; Okuhara et al., 2019), Bmps, other Tgfβ family members and their antagonists (Greene and Pisano, 2005; Nie et al., 2006a; Matsui and Klingensmith, 2014; Graf et al., 2016; Young et al., 2017), and Wnts (Alexander et al., 2014; Ji et al., 2019). Thus, it may well be that DeMyer reversed the valence of their prediction: the face may *reflect* the brain, but the brain, *via* the neural crest, *predicts* the face.

**Figure 2:**
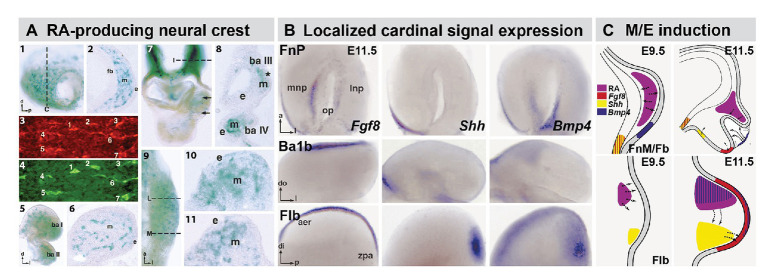
The distribution of RA producing neural crest at sites of M/E induction and its relationship to epithelia and mesenchymal sources of additional cardinal inductive signals. **(A)** Subsets of frontonasal (panels 1, 2), branchial arch (panels 5, 6), aortic arch (panels 7, 8), and forelimb bud (Flb; panels 9–11) are labeled by the βgeo6 reporter. These cells are coincident with Raldh2-expressing cells in the frontonasal mesenchyme (FnM; panels 3, 4) as well as other sites of non-axial M/E interaction. The dotted lines in panels 1, 7, and 9 indicate the approximate plane of the sections shown in panels 2, 8, 10, and 11, respectively. **(B)** *In situ* hybridization identifies local expression of cardinal inductive/morphogenetic signals *Fgf8*, *Shh*, and *Bmp4* in epithelial as well as mesenchymal domains in the frontonasal process (FnP), mandibular arch (Ba1b), and Flb. *Fgf8* and *Shh* are limited to epithelial domains in the medial nasal process (mnp) while *Bmp4* is enhanced in the lateral nasal process epithelium. *Fgf8* is found in a limited dorsal-lateral epithelial domain in Ba1b, *Shh* in a medial domain, and *Bmp4* in a dorsal medial location. In the Flb, these three cardinal signals define the apical ectodermal ridge (aer) and zone of polarizing activity (zpa), two embryologically defined signaling regions that drive limb morphogenesis and patterning (REFS). **(C)** Schematic summary of the localization and signaling interactions (arrows) of local mesenchymal and epithelial sources of RA, Fgf8, Shh, and Bmp4 in the frontonasal mass/Fb (top) and Flb (bottom) during the initial establishment of these sites of non-axial neural crest-mediated M/E induction (Embryonic day E9.5 in the mouse) and as signaling and morphogenesis moves forward (E11.5). The direction of the arrows was determined using *in vitro* mesenchymal/epithelial co-cultures or isolated explants of the epithelium or mesenchyme alone (LaMantia et al., 2000; Bhasin et al., 2003).

The distribution of the neural crest in the midgestation embryo includes several discrete accumulations of mesenchymal cells at bilaterally symmetrical locations (Figures 1B,C, 2). The neural crest-derived mesenchyme at some of these sites will contribute to special sensory organs: the frontonasal masses [olfactory epithelium (OE) and nose], the eyes (cornea, scleral, and choroidal cells), and the otic placodes (middle ear bones and epithelia); others will generate cranial skeletal elements, teeth, and cartilage (Jiang et al., 2002; Lwigale et al., 2004; Tucker and Sharpe, 2004; Balmer and LaMantia, 2005; Creuzet et al., 2005; Yoshida et al., 2008; Edlund et al., 2015; Williams and Bohnsack, 2015; Dash and Trainor, 2020). Each of these mesenchymal neural crest populations derives from a distinct anterior to posterior (A-P) location in the mesencephalic, rhombencephalic, vagal/cardiac, or trunk neural crest (Figure 1B). The somewhat surprising inclusion of the limb bud in this list of sites of early neural crest mesenchymal accumulation in the early limb bud prior to morphogenesis has been commented on in classical embryological studies (Erickson, 1985; Grim and Christ, 1993) and suggested – sometimes without comment – by additional work using molecular and genetic methods (Noakes and Bennett, 1987; Shen et al., 1997; Barlow et al., 2002; Akiyama et al., 2005; Olaopa et al., 2011; Wade et al., 2012). Our work using transgenic reporters and molecular markers for neural crest have reinforced the likely presence of neural crest in the limb bud mesenchyme (Maynard et al., 2002; Bhasin et al., 2003; Meechan et al., 2006; Rawson et al., 2010) prior to the ingression of nerves and vascular cells (Le Douarin et al., 1991; Jessen and Mirsky, 2005). Thus, subsets of neural crest cells that migrate to distinct peripheral sites of morphogenesis, including the facial primorida, bring with them a record of A-P neural tube position and presumably share aspects of molecular identity with neural progenitor cells that remain behind. These include expression and activity of *Hox* genes and related factors within distinct A-P domains.

The fates of these distal mesenchymal neural crest cells will ultimately include skeletogenic progenitors, sensory and autonomic neurons, Schwann cells, melanocytes, and in some cases vascular cells (Thomas and Erickson, 2008; Nitzan et al., 2013; Trost et al., 2013; Petersen and Adameyko, 2017). Nevertheless, during an earlier epoch of development, after migration but before terminal differentiation, they serve a distinct function. These mesenchymal neural crest cells localize sources of inductive signals directly, or *via* interactions with adjacent ectoderm (Figure 2) to drive morphogenesis and differentiation (LaMantia et al., 1993, 2000; Neubuser et al., 1997; Bhasin et al., 2003; Thesleff, 2003). These local sites of mesenchymal/epithelial (M/E) induction generate essential, bilaterally symmetric peripheral structures in all vertebrates that facilitate the organism’s interactions with its environment, as well as circuits in the central nervous system that animate these structures.

Many “cardinal” morphogenetic signals, including Shh, Bmps, and Fgfs, are expressed in epithelial domains at sites where mesenchymal neural crest accumulates in the head as well as heart and limbs (Figure 2). Their expression relies on localization of the neural crest and its capacity to secrete signaling molecules, particularly RA (Bhasin et al., 2003). Thus, the brain, *via* neural crest specified along the A-P axis of the neural tube, drives the development of facial structures, including the nose and jaws, and the neural crest from more posterior regions of the neural tube performs a similar function for the great vessels of the heart or for patterning and morphogenesis in the limbs (Figure 2). With the benefit of nearly 60 years of subsequent embryological, cell biological, molecular, and genetic observations, one can confidently revise and extend the conclusion of DeMyer et al. that “the face predicts the brain”: *the brain builds the face* – *and other non-axial bilaterally symmetric structures along the A-P axis*. This morphogenetic relationship between the brain, neural crest, and the periphery has another essential consequence: developmental coordination that integrates sensory and/or motor functions of biomechanical specializations that execute essential behaviors. Accordingly, this mechanism is a likely target for central nervous system dysfunction and related peripheral dysmorphology in multiple neurodevelopmental disorders.

## Coordinated Differentiation of a Sensory Pathway by Neural Crest: Olfactory Development

If the brain does indeed build the face and other target sites, what are the likely purposes of this construction effort? Data from my laboratory over several decades suggests that an essential purpose of brain-constructed facial primordia is to coordinate the development of peripheral sensory sites with that of their central nervous system targets. Our work on initial specification and subsequent differentiation of the primary olfactory pathway provided initial support for this hypothesis (LaMantia et al., 1993, 2000; Anchan et al., 1997). The inspiration for this hypothesis came from classical embryological experiments that demonstrated the inductive capacity of the entire olfactory placode (op), presumably both ectodermal and mesenchymal components, to induce a supernumerary limb when transplanted beneath the flank ectoderm (Balinsky, 1956). In addition, extirpation and transplantation experiments suggested that the olfactory op exerted significant inductive influence on its primary target, the anterior Fb in both the frog and the mouse (Graziadei et al., 1978; Stout and Graziadei, 1980; Graziadei and Monti-Graziadei, 1992). Finally, observations in hamster embryos suggested that RA teratogenesis at a limited period of early Fb development – after the neural crest has arrived in the anterior cranial region – results in a loss of both the OE and the OB (Shenefelt, 1972). Thus, based upon tissue-tissue interactions and the apparent involvement of a key morphogenetic signal, it seemed possible that early induction played a role in olfactory pathway development.

**Figure 3:**
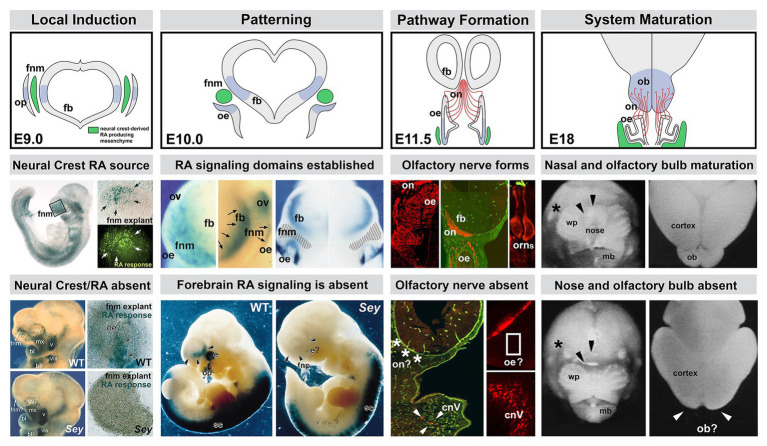
The sequence of neural crest-mediated M/E induction and its consequences for local patterning, neuronal differentiation, and initial establishment of the axon growth and targeting from the olfactory placode. The *top panels* show the stepwise initial development of the olfactory placode (op; blue shading), olfactory epithelium (OE; blue shading), and olfactory receptor neurons (ORNs) and their axons that constitute the nascent olfactory nerve (ON; red). A sub-population of mesencephalic/diencephalic neural crest cells in the FnM (green) produce RA and establish domains of RA-mediated gene expression (blue shading) in the Fb as well as the olfactory periphery. This Fb domain will generate the olfactory bulb (OB), the target of the axons from the OE *via* the ON. The *middle panels* summarize the inductive, patterning, sensory neuron differentiation and axon outgrowth, peripheral and brain morphogenetic events diagramed in the top panels. The *bottom panels* show the disruption of neural crest-mediated M/E interaction in the *Pax6* ^Sey/Sey^ mutant and its consequences for each subsequent step of initial olfactory pathway formation (top panel adapted from LaMantia et al., 1993; middle and bottom panels adapted from LaMantia et al., 1993, 2000; Anchan et al., 1997; Balmer and LaMantia, 2004; Tucker et al., 2010).

We first asked whether there was inductive signaling *via* RA that normally prefigures the establishment of the anlagen of the OE and the OB, and whether this signaling influences the initial projection of olfactory sensory afferents to their OB targets (LaMantia et al., 1993). The coordinated effects of frontonasal mesenchyme (FnM) signaling, *via* production of RA by the mesenchyme only, establish domains of RA-mediated gene expression in the cranial surface ectoderm and ventral Fb neuroectoderm. These domains are sites of the earliest neurogenesis in the cranial periphery and the Fb (Figure 3). They will eventually differentiate as the OE in the periphery and the OB in the Fb. Finally, the cranial mesenchyme apparently constrains the initial growth of olfactory receptor neuron (ORN) axons to the presumptive OB as well as the morphogenesis of the bulb itself (Whitesides and LaMantia, 1996; Tucker et al., 2006). These inductive events are critically dependent on the migration of primarily mesencephalic neural crest cells into the most anterior aspect of the embryo as the anterior neural tube closes (Serbedzija et al., 1992; Osumi-Yamashita et al., 1994). These cells constitute the FnM interposed between the anterior surface ectoderm and the ventral neuroepithelium of the nascent prosencephalic vesicle.

Substantial attention has been given to the fates of neural crest cells that constitute the frontonasal as well as the branchial arch and lateral cranial mesenchyme (Noden, 1988; Gross and Hanken, 2008; Weston and Thiery, 2015). Ultimately, subsets of these cells will become progenitors for facial and pharyngeal bones and cartilage including those of the nose. Others will contribute to the cranial sensory ganglia (D’Amico-Martel and Noden, 1983; Freyer et al., 2011; Karpinski et al., 2016; Thiery et al., 2020). Still, others will constitute populations of melanocytes or vascular cells (Miller et al., 2017; Vandamme and Berx, 2019). Prior to acquiring those fates, however, subsets of these cells serve transient but distinct developmental functions: they provide molecular signals to adjacent tissues to elicit focally patterned gene expression and drive local cellular differentiation. This role for neural crest has been established for initial patterning and differentiation of cranial musculature and vasculature from mesodermal progenitors (Hill et al., 2015; Ziermann et al., 2018). Similar neural crest-mediated signaling mechanisms, prior to fate restriction and differentiation of these cells, also coordinate cranial peripheral and central nervous system development.

Focal-inductive signaling mediated by cranial neural crest prefigures and likely drives specification of peripheral and central “olfactory” progenitors for distinct neuronal fates. The neural crest cells of the FnM fulfill this role in at least three ways (Figure 3): first, they provide signals that pattern additional signaling centers in adjacent epithelia that then drive neuronal as well as skeletal differentiation (Bhasin et al., 2003; Marcucio et al., 2005). Second, they signal directly to neural progenitors to modulate division, migration, or fate in the OE as well as Fb precursors that generate OB interneurons (LaMantia et al., 1993; Anchan et al., 1997; Whitesides et al., 1998; Tucker et al., 2006, 2008). Third, they provide molecular guidance cues to growing ORN axons (Whitesides and LaMantia, 1996; LaMantia et al., 2000; Rawson et al., 2010).

Our data indicates that only a subset of FnM neural crest cells produce RA, based upon *in vitro* transcriptional “indicator” assays with a monolayer of heterologous cells substituting for endogenous target epithelia (Whitesides et al., 1998; LaMantia et al., 2000; Bhasin et al., 2003). Moreover, *in vivo*, the activation of a similar RA signaling reporter in subsets of presumed RA-responsive cells or expression of RA-responsive genes in the OE and Fb (Figure 3) indicates that the neural crest-derived FnM provides a local source of RA to drive expression of downstream genes in cranial ectodermal and neural tube domains that eventually generate OE and OB neurons (LaMantia et al., 1993, 2000; Whitesides et al., 1998; Rawson and LaMantia, 2007). The RA signaling capacity of neural crest mesenchymal cells in the frontonasal processes and other sites of non-axial M/E apposition reflects local expression and activity of RA synthetic enzymes, including Raldh2 and Raldh3 (Berggren et al., 1999; Haselbeck et al., 1999; Niederreither et al., 1999, 2002, 2003; Mic et al., 2000; Suzuki et al., 2000; Mey et al., 2001). RA synthesis and activity can be further influenced by expression of retinoid binding proteins and differential expression and activity of RA receptors and binding proteins in adjacent neural crest or target epithelial cells (Perez-Castro et al., 1989; Maden et al., 1991; Ruberte et al., 1991; Gustafson et al., 1993; Lohnes et al., 1994; Whitesides et al., 1998). The expression of many of these molecules persists throughout through adulthood and may influence ongoing ORN genesis and differentiation in the adult OE and OB (Whitesides et al., 1998; Thompson Haskell et al., 2002; Haskell and LaMantia, 2005; Hagglund et al., 2006; Peluso et al., 2012; Paschaki et al., 2013; Micucci et al., 2014; Login et al., 2015).

## A Watch on the “Rhine”: Rhinencephalic Mutants, M/E Induction, and Olfactory Development

Genetic analysis reinforced the essential contribution of M/E interactions between neural crest and additional local epithelial or mesenchymal cells in the initial assembly of the primary olfactory pathway from nose to brain. We selected four fully or partially arhinencephalic mutants in which initial olfactory pathway morphogenesis is disrupted: the *Pax6* “*Small Eye*” mutation (Hill et al., 1991) in which both the OE and OB fail to form (Grindley et al., 1995; Anchan et al., 1997; Jimenez et al., 2000); The *Gli3 Extra Toes^J^* mutation (Schimmang et al., 1992; Hui and Joyner, 1993) where the OE differentiates, but olfactory axons fail to enter the Fb in which the OB is absent (Sullivan et al., 1995; LaMantia, 1999; Balmer and LaMantia, 2004; Taroc et al., 2020); the *Shh* null mutant (Chiang et al., 1996; Ishibashi and McMahon, 2002; Hayhurst and McConnell, 2003), which is a model for the most extreme cases of holoprosencephaly or arhinencephaly – the clinical dysmorphologies that initially inspired DeMyer et al. to conclude that “the face predicts the brain” – and the *Fgf8* ^Neo^ hypomorphic mutant in which ORN neurogenesis and OB morphogenesis is disrupted (Meyers et al., 1998; Kawauchi et al., 2005; Tucker et al., 2010). In each case, frontonasal and Fb M/E interactions are compromised with morphogenetic as well as cellular consequences for olfactory pathway development.

In the *Pax6* ^−/−^ mutant, the OB and OE are absent, and the anterior snout, maxilla, and mandible are either absent or dysmorphic (Grindley et al., 1995; Anchan et al., 1997; Enwright and Grainger, 2000). In this mutant, RA signaling is abolished in domains that generate the OE and OB due to the failure of the RA-producing neural crest to migrate into the frontonasal region. The absence of the neural crest derived mesenchymal cells, and the failure of placodal and ventral Fb RA-mediated M/E signaling prefigures the morphogenetic failure of both the OE and the OB (Figure 3). The residual mesenchyme from *Pax6* ^Sey/Sey^ cannot support olfactory neuron differentiation in WT pre-placodal ectoderm (LaMantia et al., 2000). Nevertheless, the capacity for RA responsiveness in both the cranial ectoderm and Fb neuroectoderm remains; however pharmacological activation of this responsiveness by exogenous RA fails to elicit recognizable differentiation of olfactory structures or their constituent neurons (Anchan et al., 1997).

The three additional mutants – *Gli3*, *Shh*, and *Fgf8* – provide further support for a primary role of M/E interactions that engage neural crest and adjacent epithelia in olfactory pathway development. In *Gli3* ^XtJ^ homozygotes, the Fb neuroepithelium is refractory to RA signaling despite local production of RA by neural crest-derived frontonasal mesenchymal cells and the OB does not form (LaMantia, 1999). Axons from differentiated ORNs grow into the apparently normally patterned FnM; however, they mostly fail to enter the undifferentiated Fb, with the exception of a few misrouted axon fascicles that manage to fenestrate the Fb basal lamina (Whitesides and LaMantia, 1996; Balmer and LaMantia, 2004). *Shh* ^−/−^ mutants have a fused proboscis, and a single fused OE in which ORNs differentiate. This OE appears to be primarily “lateral” in its identity, surrounded by FnM that is also “lateral” based upon restricted expression of neural crest-associated markers, including Pax7 (Mansouri et al., 1996; Monsoro-Burq, 2015). In the *Shh* ^−/−^ mutant, as in *Gli3* ^−/−^, where RA signaling (LaMantia, 1999; Maynard et al., 2013) as well as Shh signaling (Tole et al., 2000; Stamataki et al., 2005) is disrupted, ORN axons grow toward, but fail to enter, the dysmorphic Fb (Balmer and LaMantia, 2004).

Finally, in *Fgf8* ^Neo/Neo^ mutants, the expression levels of one of the inductive targets of the RA-producing FnM, *Fgf8* (see Figure 2), are substantially diminished (Meyers et al., 1998). ORN frequency in these mutants is diminished and their distribution altered due to disrupted proliferative capacities and neurogenic potential of distinct ORN precursor classes (Tucker et al., 2010). These changes parallel the disruption of OB differentiation in the dysmorphic Fb of *Fgf8* ^Neo/Neo^ mutants (Meyers et al., 1998; Kawauchi et al., 2005). These observations suggest that in the absence of downstream signaling molecules like Fgf8, whose local sources in the cranial ectoderm or Fb are patterned and maintained by neural crest-derived mesenchymal RA-producing cells (Bhasin et al., 2003), morphogenesis and assembly of the olfactory pathway fails. Thus, observations in WT and mutant embryos define the central role of neural crest in local M/E interactions, especially those mediated by RA signals provided by neural crest mesenchymal cells, for the coordination of morphogenesis and subsequent connectivity of the olfactory system during early stages of Fb development.

## Other Cranial Neural Crest Cells and Other Cranial Nerves

The role of the neural crest in the patterning or differentiation in other placodal derivatives that establish additional cranial sensory specializations is less clear. In contrast to early “inductive” events that specify cranial sensory pre-placodal ectoderm (Jacobson, 1963; Maier et al., 2014; Moody and LaMantia, 2015; Thiery et al., 2020), the subsequent interactions between each of the placodes, once specified, and neural crest cells – aside from those that influence differentiation of the embryonic OE – are less well understood. Embryological experiments suggest that the neural crest is not essential for the initial placode induction (Begbie et al., 1999; Haworth et al., 2004). Thus, the initial specification of the preplacodal ectoderm that will generate neural progenitors for the olfactory, trigeminal, and epibranchial/sensory placodes, as well as the lens and otic placode, relies upon planar signals and transcriptional effectors within the cranial ectoderm, as well as extrinsic signals from the lateral head mesoderm and prechordal mesendoderm (Hintze et al., 2017), and this process is coincident with the specification of the neural crest at the neural plate border zone (Rogers et al., 2012; Saint-Jeannet and Moody, 2014).

In contrast, post-migratory neural crest cells have distinct intermediate functions as well as terminal fates during initial morphogenesis of the eye, ear, and cranial ganglia. In the developing eye, neural crest-derived mesenchymal cells establish a local source of Tgfβ ligands that suppresses lens fate in presumptive lens epithelium, a placodal derivative (Grocott et al., 2012). In addition, RA-mediated signaling between the optic cup epithelium (neural tube-derived) and the neural crest-derived mesenchyme surrounding the eye is essential for ocular morphogenesis (Creuzet et al., 2005; Matt et al., 2005; Bailey et al., 2006). Less is known about the role of neural crest in signaling during otic placode differentiation. Neural crest cells contribute to the middle ear as well as generating glial cells that ensheathe axons from the acoustic/spiral ganglion (Chapman, 2011; Thompson and Tucker, 2013; Ritter and Martin, 2019). There is also some evidence that neural crest cells contribute to the inner ear (Freyer et al., 2011). Signaling *via* RA and Fgf8 from partly defined sources contributes to A-P patterning of the otic placode ectoderm, which is presumably the source of sensory neurogenic precursors, as well as the periotic mesenchyme which generates middle ear bones and epithelia (Frenz et al., 2010; Bok et al., 2011; Nakajima, 2015). The otic placode epithelium, presomitic, somatic mesoderm, and periotic mesenchyme have been suggested as RA sources during initial otic vesicle patterning; however, the contribution of neural crest to periotic mesenchyme has not been considered in the context of signaling prior to differentiation. Thus, the role of neural crest derived M/E interactions in the eye and ear, vs. the nose, remains uncertain.

**Figure 4:**
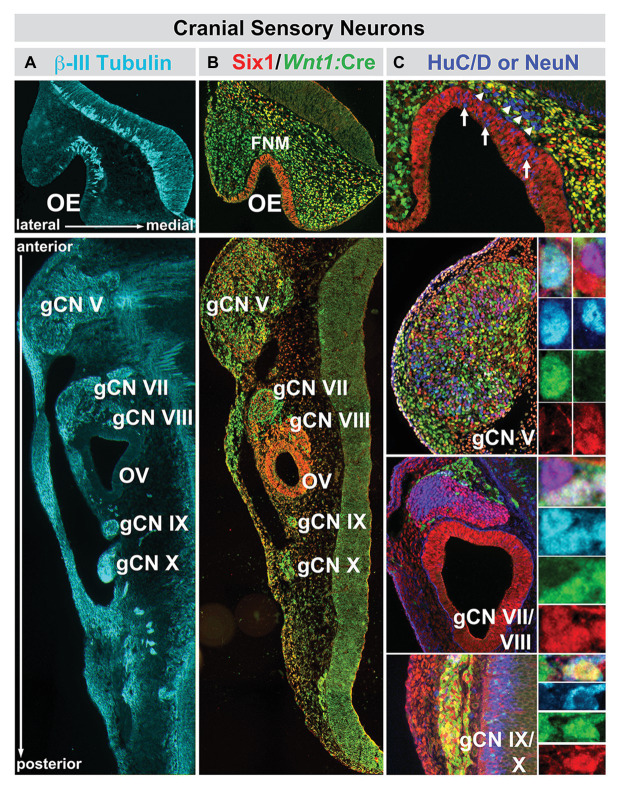
The relationship between nascent cranial sensory neurons, ectodermal placode, and neural crest-derived neural progenitors and neuroblasts in the cranial sensory ganglia at midgestation (E10.5) in the mouse. **(A)** The neuronal microtubule protein βIII-tubulin is expressed in newly generated ORNs (OE, top panel) as well as cranial sensory neurons in the trigeminal (gCN V), facial (gCN VII), spiral (gCN VIII), glossopharyngeal (gCN IX), and vagal (gCN X) cranial nerve ganglia and their axons that extend toward central (OE, gCNV, VII, VIII, IX, and X) as well as peripheral (gCNV, VII, VIII, IX, and X) targets. **(B)** Six1 (red), a marker of placode-associated cells and *Wnt1:Cre* recombination-mediated expression of a conditional GFP reporter allele (green), shows the relationship between placode-associated cells and neural crest-derived cells in the OE, FnM and cranial ganglia. Cells in the OE are labeled exclusively by Six1. Cells in the FnM are uniformly labeled by the *Wnt1:Cre* reporter, but a subset of them in the lateral nasal process also expresses Six1. Each of the cranial nerve ganglia, except for gCN VIII, is composed of primarily Six1-expressing placode-derived cells. The mesenchyme between the cranial nerve ganglia and the hindbrain at this stage of development has cells that express Six1 as well as the *Wnt1:Cre* reporter, as is the case for the cranial epithelium in the periphery. **(C)** Relationship between Six1-expressing, Wnt1:Cre reporter-expressing, and HuC/D-expressing cells in the OE and cranial nerve ganglia. In the OE, HuC/D-expressing newly generated neurons (blue) are scattered through the epithelium and have downregulated Six1 (arrows). In addition, there is a population of HuC/D expressing neurons (arrowheads) in the FnM that have also downregulated Six1 and are not labeled by the *Wnt1:Cre* reporter. These cells are most likely the GnRH-expressing neurons that migrate from the OE to enter the ventral Fb along newly extending ORN axons at this stage of development. In gCN V, gCN VII, and gCN IX/X, HuC/D-expressing neurons are coincident with cells labeled by Six1, the *Wnt1:Cre* reporter, or both (adapted from Karpinski et al., 2016).

We have begun to assess interactions between neural crest and placodal cells underlying development of cranial somatosensory ganglia. The dual origin of cranial ganglion sensory neurons, as well as their divergent fates – primarily mechanoreceptive for placode descendants, nociceptive for those from the neural crest (D’Amico-Martel and Noden, 1983) – is well established, and our analyses in the mouse (Karpinski et al., 2016; Maynard et al., 2020a; Motahari et al., 2020) confirmed and extended earlier studies. Using transcriptional lineage tracing, we identified diversity within the neural crest population (Figure 4). Neural crest-associated progenitors in all cranial ganglia include a population derived from a *Wnt1* expressing domain in the dorsal/alar hindbrain (McMahon et al., 1992; Chai et al., 2000), and a population apparently not derived from this region that nevertheless expresses established neural crest markers including Foxd3 and Sox10. The proportions of these populations, placode-derived cells and each of the two molecularly distinct neural crest cell classes, are statistically similar in most cranial ganglia (Karpinski et al., 2016). In contrast, placode-derived populations predominate in the “special sense” organs – the OE and the inner ear. There is some uncertainty, however, over the contribution of the neural crest to initial populations of OE progenitors and early generated ORNs (Forni et al., 2011; Karpinski et al., 2016). It is also possible that at later fetal stages and in the adult OE, neural crest-derived progenitors can generate ORNs (Katoh et al., 2011; Suzuki et al., 2013). For auditory peripheral receptors and relay neurons, there is some evidence that subsets of sensory receptors (outer and inner hair cells) in the inner ear, as well as sensory relay neurons in the spiral ganglion (Cranial Nerve ganglion VIII; Figure 4), are derived from neural crest progenitors (Freyer et al., 2011).

Nevertheless, the four cranial ganglia responsible for the somatosensory regulation of orofacial sensory-motor integration: trigeminal (CNgV), facial (CNgVII), glossopharyngeal (CNgIX), and vagal (CNgX) are mosaics of substantial populations of placode-and neural crest-derived cells that condense with the cranial mesenchyme sometime after the anterior neural tube closes in most vertebrate embryos. Thus, for all cranial sensory neurons or the sensory organs in which they are found, neural crest-derived cells accumulate, interact with cranial ectodermal placodal cells, and either induce supporting structures or special sensory neurons or coalesce to form cranial ganglia after the translocation of placodal cells into the cranial mesenchyme. This confluence of neural crest and cranial ectoderm indicates that at the earliest stages of development, the fates of cells that will constitute the face and those that will comprise neural circuits in the peripheral and central nervous system that innervate the face (Cordes, 2001) are closely related. Parallel work in the spinal cord and its musculoskeletal or visceral targets suggests that coordination between early neural tube and peripheral patterning is essential for establishing appropriate neural circuits to control limb and visceral targets (Philippidou and Dasen, 2013). Thus, like the development of the spinal cord and limb, the hindbrain and the face may reflect a singular developmental program that coordinates peripheral structures and the neural circuits that control these structures.

## Beyond the Face? a Genetic Disorder with Face, Limb, Heart, and Fb Anomalies

The coincidence of so-called minor physical anomalies – mild to severe malformations of craniofacial structures, including ears, eyes, and noses and the limbs – as well as increased coincidence of cardiovascular malformations in a number of clinically diagnosed behavioral syndromes like schizophrenia and autism or multiple genetic neurodevelopmental disorders (Tripi et al., 2008; Compton et al., 2011; Delice et al., 2016; Myers et al., 2017), led to an additional test of our central hypothesis: the coordinated regulation of neural crest-mediated M/E interaction may be central to optimal morphogenesis at each of the sites of non-axial induction, including limbs, face, heart, and Fb. Accordingly, this mechanism may be uniformly disrupted in disorders that include minor physical anomalies, cardiovascular malformations, and Fb developmental disruption – reflected in complex behavioral deficits that define clinically diagnosed neurodevelopmental disorders like schizophrenia and autistic spectrum disorder as well as several genetic neurodevelopmental syndromes.

We selected the microdeletion disorder 22q11.2 Deletion Syndrome (22q11DS), also known as DiGeorge or Velocardiofacial syndrome, to evaluate our hypothesis. 22q11DS is a global developmental disorder whose phenotypic spectrum includes highly penetrant cardiovascular malformations, as well as craniofacial anomalies, mild limb and digit anomalies, and a high frequency of behavioral difficulties that resemble clinically diagnosed neurodevelopmental disorders, including schizophrenia, autistic spectrum disorder, and attention deficit disorder, accompanied by altered brain morphology and function (Schneider et al., 2014; McDonald-McGinn et al., 2015; Rogdaki et al., 2020). 22q11DS, as the name suggests, is not caused by a single loss-of-function mutation but deletion of a limited number of genes: minimally 32 in humans (Morrow et al., 2018) and their subsequent approximately 50% diminished expression (Meechan et al., 2006; Maynard et al., 2008, 2013, 2020a). There is a high level of conservation of these genes, as a colinear set, across multiple vertebrates, including the mouse, in which 28 of the 32 minimally critically deleted genes are found adjacent to one another on murine Chromosome 16 (Meechan et al., 2015). There is remarkable homology of the colinear set of 22q11-deleted genes in multiple species, and their cellular, developmental, and homeostatic functions in a broad range of cells, tissues, and organs appear to be similar in most vertebrates – and even some invertebrates – analyzed thus far (Meechan et al., 2015; Motahari et al., 2019). A key aspect of 22q11 gene function across these species may be the apparent relationship to neural crest, and non-axial M/E signaling at midgestation, and its consequences for subsequent morphogenesis and neural circuit development.

## Location, Location, Location: Restricted Expression and Activity of 22q11 Genes at M/E Sites

**Figure 5:**
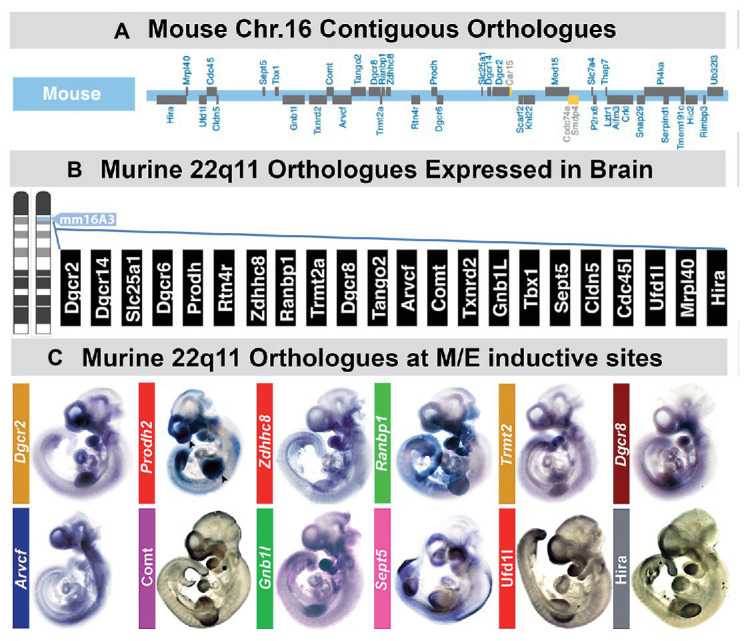
A large subset of mouse orthologues of the genes on human Chr. 22 deleted in DiGeorge/22q11 Deletion syndrome (22q11DS) are expressed in the developing or adult brain as well as sites of neural crest-mediated M/E induction at midgestation. **(A)** The location on mouse chromosome 16 of 28/32 orthologues of the genes in the minimal critical deleted region of human chromosome 22 whose heterozygous deletion causes 22q11DS. **(B)** PCR, *in situ* hybridization, immunoblotting, and immunlocalization identify expression of 22 of the 28 murine 22q11 orthologues in the developing and adult mouse brain. **(C)** Multiple 22q11 orthologues are expressed uniformly at sites of neural crest-mediated M/E induction as well as in the nascent central nervous system in the midgestation mouse embryo (E10.5). The purple-blue label shows *in situ* hybridization labeling of mRNA for several 22q11 genes at these sites, and the brown label shows the localization of proteins encoded by three of the deleted genes (panel A, B, adapted from Meechan et al., 2015; panel C adapted from Motahari et al., 2019).

The first question we asked was whether one, two, or a few of the genes deleted in 22q11DS were expressed at sites of non-axial M/E interaction where 22q11DS phenotypes will eventually arise: limb buds, cardiac-related/pharyngeal arches, craniofacial pharyngeal arches, and the cranial or FnM (Maynard et al., 2002, 2003). Rather than a few 22q11 deleted genes, we found that 21/28 are expressed selectively at these sites based upon qPCR analysis in micro-dissected samples of each M/E inductive site as well as whole mount *in situ* or immunolabeling (Figure 5). None of the 22q11 genes is known to selectively alter neural crest specification or migration (Motahari et al., 2019). Instead, many of these genes, including several candidates for specific 22q11DS phenotypes, seem to modulate either local patterning, differentiation, or signaling. Complete loss of function mutations of some of these genes lead to substantial dysmorphology at several sites of non-axial M/E induction (Scambler, 2010; Paronett et al., 2015; Motahari et al., 2019), while heterozygous deletion, usually in the context of broader 22q11 gene deletion, leads to variable dysmorphology or dysfunction in a variety of organ systems.

Two additional observations reinforce the conclusion that 22q11 genes, as a group, contribute to the local regulation of M/E interactions at sites of non-axial induction. First, disrupted signaling, particularly *via* RA, Fgfs, Bmps, or Wnts – all implicated in non-axial M/E signaling and morphogenesis – can recapitulate, at least partially, some of the phenotypes associated with 22q11DS (Frank et al., 2002; Bachiller et al., 2003; Vermot et al., 2003; Aggarwal et al., 2006; Huh and Ornitz, 2010; Guo et al., 2011). Several of these signaling pathways are sensitive to 22q11 gene dosage, based upon dysmorphic phenotypes or altered patterns and levels of gene expression in mouse models. There are genetic interactions between diminished dosage of 22q11 genes, particularly *Tbx1*, a 22q11 gene for cardiovascular and pharyngeal arch phenotypes, and the RA, Shh, Fgf, and Bmp signaling pathways (Garg et al., 2001; Ryckebusch et al., 2010; Maynard et al., 2013). Our data suggests that interactions between the 22q11 genes, RA and Shh signaling, are enhanced by full 22q11 deletion compared to that seen in *Tbx1* ^+/−^ mutants. Together, these observations suggest reciprocal local regulation for 22q11 gene dosage and cardinal signaling pathways at sites of non-axial accumulation of neural crest mesenchymal cells, neural crest-mediated M/E inductive interactions, and downstream morphogenetic mechanisms.

22q11DS has been classified as a neural crest disorder or “neurocristopathy” based upon the coincidence of cardiovascular and craniofacial phenotypes (Walker and Trainor, 2006; Vega-Lopez et al., 2018). The available evidence, however, indicates that, at least for the cardiovascular malformations, the differentiation capacity of the neural crest is not substantially targeted by 22q11 deletion or heterozygous loss of function of *Tbx1*, a 22q11 gene considered a candidate for the cardiovascular phenotype whose expression and activity is limited to the pharyngeal mesoderm and endoderm (Kelly et al., 2004; Arnold et al., 2006; Aggarwal et al., 2010). Instead, 22q11DS phenotypes may result from altered neural crest-mediated interactions with mesodermal or endodermal targets that express *Tbx1*. Our evidence suggest that a significant portion of the 22q11DS phenotypic spectrum reflects the coordinated expression of multiple 22q11 genes and their dosage-sensitive influence on neural crest-mediated M/E induction beyond that of *Tbx1* (Maynard et al., 2013, 2020a; Karpinski et al., 2014; Motahari et al., 2020). These 22q11 genes have reciprocal regulatory interactions with cardinal inductive signaling pathways critical for optimal M/E induction at each of the phenotypic sites. When these interactions are disrupted by diminished dosage of multiple 22q11 genes, altered M/E induction results in a sequence of pathogenic changes that contribute to the phenotypic spectrum associated with 22q11DS.

## Other Faces, Other Brains: an Unmapped Monogenic Disorder with Face, Limb, and Behavioral Phenotypes

**Figure 6:**
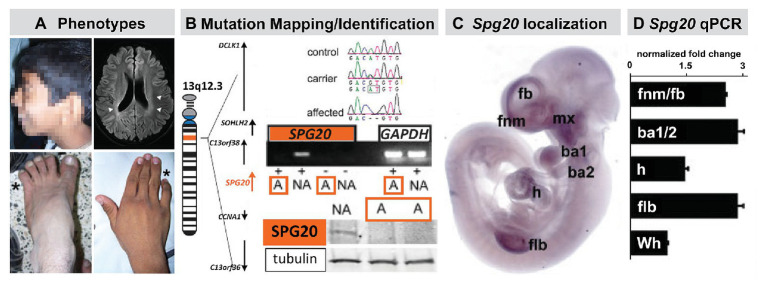
The mutant gene in a rare monogenic disorder characterized clinically by craniofacial, limb, and Fb anomalies is initially expressed focally and maximally at sites of neural crest-mediated M/E induction. **(A)** Craniofacial, brain, hand (forelimb), and foot (hindlimb) anomalies in a 19-year-old male. This individual also had developmental delay, poor academic performance, and poor language skills from an early age. **(B)** Mapping and confirming the causal mutant gene for this Mendelian, monogenic disorder. The mutant gene SPG20, is a microtubule-interacting trafficking molecule involved in multiple signaling and metabolic cellular processes. The mutation in this Omani pedigree is a novel *SPG20* two base pair deletion mutation that results in undetectable expression of *SPG20* in fibroblasts from affected individuals in the pedigree, as well as undectectable Spartin protein expression. **(C)** Localization of *Spg20*, the murine orthologue of *SPG20* by *in situ* hybridization in an E10.5 mouse embryo, shows focal, selective expression at sites of neural crest-mediated M/E induction, including FnM and Fb, the maxillary process (mx), and as well as the nascent mandibular process (ba1), the hyoid process (ba2), the heart (h), and Flb. **(D)** qPCR in microdissected frontonasal mass/Fb, branchial arches, h and Flb confirms enhanced expression of *Spg20* at these M/E inductive sites. These expression levels, especially for the fnm/fb, ba1/2, and flb, are substantially elevated above the expression level detected in the whole E10.5 embryo (wh; adapted from Manzini et al., 2010).

The relationship between 22q11 genes and cardinal signaling pathways at sites of M/E interaction and pathogenesis of 22q11DS phenotypes suggests that mutations of additional genes that influence early neural crest-mediated M/E induction may result in craniofacial and brain anomalies in additional human genetic developmental disorders. To evaluate this possibility, we identified an apparently monogenic, homozygous autosomal recessive, human genetic disorder in a consanguineous pedigree (Manzini et al., 2010). Affected individuals had craniofacial and limb dysmorphology as well as Fb-related behavioral disruption. We mapped and identified the mutated gene and then assessed the timing and localization of expression of this gene during development in both human and mouse (Figure 6).

The proband for this study was an affected male with craniofacial and limb morphological/skeletal anomalies, dysarthria, developmental delay, and intellectual/cognitive impairment. Unaffected siblings had none of these phenotypes (Figure 6). In addition to these “core” morphological and behavioral features, this disorder was accompanied by spasticity, and some evidence of neurological degenerative change over the lifespan. The causal mutation for this disorder in the Omani pedigree was a novel variant of the *SPG20* gene (Figure 6) that encodes a protein called Spartin. Mutations in *SPG20* had been previously linked to Troyer Syndrome, a Hereditary Spastic Paraplegia variant identified in Amish pedigrees in which craniofacial phenotypes were not reported (Patel et al., 2002). Spartin is a microtubule interaction/intracellular trafficking-related protein thought to be involved in a range of cellular functions including microtubule dynamics, cytokinesis, endosome trafficking, mitochondrial integrity, and signaling *via* EGF and Bmps (Bakowska et al., 2007; Renvoise et al., 2010, 2012; Nahm et al., 2013). The question that emerged was whether the craniofacial morphological anomalies, overbite, hypertelorism, expanded philtrum, low set, enlarged pinnae, and the hand and foot skeletal anomalies, might reflect early morphogenetic disruption due to altered M/E interaction vs. subsequent consequences of neurodegenerative mechanisms underlying progressive spasticity.

An apparent answer to this question emerged from a quantitative expression analysis in human brain and a parallel analysis in the mouse brain as well as the early mouse embryo (Figure 6). We found that *SPG20* is expressed at varying levels in distinct regions of the adult human brain and at a comparatively higher level in the fetal human brain. While this does not discount functional significance for Spartin function in the mature human brain, it indicates that its expression is neither ubiquitous nor robust. Instead, it suggests a role for Spartin in neural development. We replicated these observations in parallel regions of the mouse brain at parallel ages; however, in the mouse, we were also able to compare *Spg20* expression levels in distinct brain regions with those in the early embryo. We found that *Spg20* is maximally expressed in the whole mouse embryo at midgestation – E10 – at relative levels far greater than any reached in the postnatal developing or mature brain (Manzini et al., 2010). We then assessed regional localization in midgestation embryos in two ways: whole embryo *in situ* hybridization to assess spatial localization and qPCR in microdissected limb buds, branchial arches, hearts, and frontonasal mass/Fb: sites of M/E interaction that share mechanistic and morphogenetic properties (Figure 6). We found selective spatial expression of *Spg20* in the mesenchyme and epithelium of the limb buds, aortic and branchial arches, frontonasal mass (most likely the mesenchyme interposed between the Fb neuroepithelium and surface ectoderm), and Fb. There was limited expression in the hindbrain and no label above background in the spinal cord. qPCR analysis showed that the expression levels of *Spg20* were highest in microdissected samples of limb buds, branchial arches, and frontonasal mass/Fb from E10.5 embryos.

Thus, there is a maximal expression of a novel gene at sites of neural crest M/E induction, and mutation of this gene results in craniofacial and limb morphogenetic disruption, as well as developmental delay and cognitive deficits, presumably due to altered brain development. Thus, consistent with the assertion that “The Face Predicts the Brain,” a combination of facial and brain phenotypes in this monogenic disorder predicted the pattern and schedule of expression and perhaps the activity of a single gene. These data suggest that Spartin may contribute to early non-axial morphogenetic mechanisms that depend upon coordinated neural crest-mediated M/E induction, including craniofacial, limb, and early Fb development.

## Putting it Together: Face, Brain, and Behavior

The predictive relationship between the face and the brain, extended to the limbs and the heart, provides a foundation to consider how development of neural circuits that organize distinct behaviors and peripheral structures that execute these behaviors might be coordinated. Such coordination may be facilitated by the ambassadorial signaling capacity of the A-P specified neural crest from distinct regions of the neural tube where related circuits will differentiate. The relationship between A-P patterning of the hindbrain, the hindbrain neural crest, the craniofacial primordia, and the cranial nerves has been assessed in the context of A-P signaling and transcriptional regulation including that *via* Hox genes and other regulators of early axial patterning (Wilkinson, 1993; Parker and Krumlauf, 2020) or for their role in coordinating the differentiation of intrinsic brainstem neural circuits (Gavalas et al., 2003; Narita and Rijli, 2009; Di Bonito and Studer, 2017). Less attention has been paid to the integration of parallel development of craniofacial structures, cranial sensory and motor nerves, and neural circuits that coordinate essential oro-facial motor behaviors. We, therefore, sought to define a distinct behavior whose neural control and biomechanical execution might be facilitated by coordinated development of the face and brain *via* neural crest-mediated signaling.

**Figure 7:**
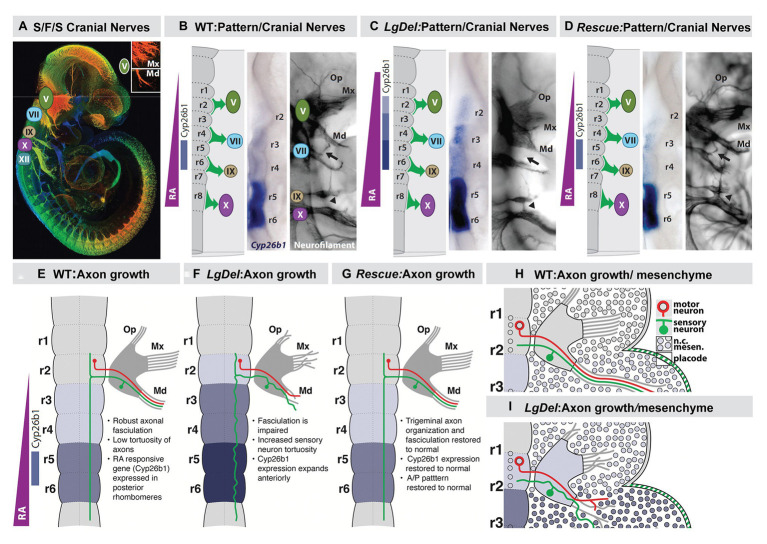
Early disruption of hindbrain patterning alters anterior cranial nerve differentiation, prefiguring anomalous oropharyngeal sensory/motor function that likely contributes to suckling, feeding, and swallowing (S/F/S) difficulties in early post-natal *LgDel* mouse pups who carry a heterozygous deletion of the 28 murine orthologues of the genes deleted in 22q11DS. **(A)** The five cranial nerves that contribute to sensory/motor control of S/F/S have begun to differentiate by E10.5 in the mouse. In this preparation, they have been immunolabeled in the whole by the early marker for neuron and axons, βIII-tubulin, and visualized in a high-resolution confocal image in which embryo volume/depth is color coded, with warm colors representing structures close to the viewer and cooler colors representing those deeper in the embryo. The inset shows the multiple small axon fascicles that characterize the maxillary branch (Mx) of the trigeminal nerve (V) and the single fascicle of axons that forms as the mandibular branch in typically developing WT embryos. **(B)** The A-P array of S/F/S contributing cranial nerves is prefigured in E9.5 embryos by a gradient of RA-signaling that distinguishes posterior (r5,6) from anterior (r2,3) rhombomeres in the developing hindbrain. This posterior RA-dependent patterning, as well as opposing anterior signaling *via* Fgfs and Wnts, specifies the precursors of the cranial sensory neurons and hindbrain motor neurons that then differentiate as the cranial nerves within 24 h. **(C)** In *LgDel* E9.5 embryos, the gradient of RA signaling is enhanced in and shifted beyond posterior rhombomeres; it now elicits RA-regulated gene expression in anterior rhombomeres. Within a day, anterior cranial nerves, V (trigeminal) and VII (facial) are dysmorphic. The multiple axon fascicles normally seen in the Mx of V are diminished, the mandibular branch is similarly hypotrophic, and the facial nerve (VII) lacks its nascent anterior branch (arrow). In addition, the posterior cranial nerves IX (glossopharyngeal) and X (vagus) have either small axonal anastomoses (arrowhead) or in extreme cases are fused. **(D)** When RA signaling levels are diminished genetically by heterozygous deletion of the RA synthetic gene *Raldh2* in *LgDel* embryos (“Rescue”), the pattern of RA-dependent gene expression in the anterior rhombomeres returns to that seen in the WT. In parallel, initial differentiation of the nascent trigeminal and facial nerve is restored to the WT state. The ophthalamic (Op), Mx, and mandibular branches of the trigeminal nerve (V) extend toward their targets as in the WT with similar degrees of fasciculation. The facial nerve branches appropriately (arrow). The fusion of the posterior cranial nerves IX and X persists, most likely because this reflects the disrupted differentiation of cardiovascular targets due to Tbx1 heterozygous deletion, independent of hindbrain RA-dependent A-P patterning. **(E–H)** Schematics of the relationship between RA-dependent hindbrain patterning and the growth and trajectory of individual trigeminal motor and sensory axons in the WT embryo. Individual trigeminal motor axons, as well as primarily placodal derived trigeminal sensory axons, respond differently as they interact with neural crest derived mesenchymal substrates in the periphery whose A-P identity has been presumably altered by enhanced RA signaling in the anterior rhombomeres.

The coordination of cranial sensory placode differentiation and that of brain targets, best exemplified by the development of the olfactory pathway, suggests the integrated development for neural circuits that relay and represent cranial “special” sensory information in one direction: from the periphery to the central nervous system. This information is further integrated by Fb “association” circuits to generate complex representations and behaviors from multi-modal sensory input (Sosulski et al., 2011; Uchida et al., 2014). In contrast, we sought to identify a behavior for which sensory inputs and motor function were more closely aligned and more precisely associated with peripheral craniofacial structures, independent, at least initially (Muscatelli and Bouret, 2018), of additional integration in Fb association circuits. One essential behavior emerged as a likely candidate: suckling, feeding, and swallowing (S/F/S; LaMantia et al., 2016; Maynard et al., 2020b). This fundamental, innate behavior shared across all mammals relies upon sequential sensory inputs and motor commands from cranial sensory and motor nerves in an approximate A-P order (Figure 7). This integrated sensory motor information subsequently activates distinct craniofacial structures to execute the biomechanical operations that permit optimal S/F/S from birth onward. Thus, S/F/S may represent a behavior whose neural and biomechanical bases reflect the predictive relationship between the face and the brain – or the brain and the face.

To evaluate this relationship, we once again began with the genetics of human developmental disorders and their consequences for morphological and behavioral disruption. The incidence of S/F/S difficulties from birth through early childhood – collectively referred to as pediatric dysphagia – is significantly elevated in genetic developmental syndromes as well as children with clinically diagnosed behavioral neurodevelopmental disorders (Berlin et al., 2011; Kleinert, 2017; Robertson et al., 2017; Bianco and Rota, 2018; Maynard et al., 2020b; Nordstrom et al., 2020), including infants and toddlers with 22q11DS (Eicher et al., 2000). Craniofacial and brain anomalies characterize all of these developmental disorders, including 22q11DS. If S/F/S is the result of coordinated neural and craniofacial development *via* neural crest-dependent signaling, genetic lesions that underlie developmental disorders should disrupt this process. This would indicate a predictive relationship between the face and brain in optimal circumstances and pathologic consequences when that relationship is disrupted in clinically or genetically diagnosed developmental disorders.

## Swallow Hard: Does the Face Predict the Brain and Behavior for S/F/S?

To assess whether distinct behavioral capacities reflect the predictive relationship between the face and the brain, we asked whether disrupted coordination of craniofacial and neural circuit differentiation in the *LgDel* mouse model of 22q11DS (Merscher et al., 2001; Meechan et al., 2015) might result in divergent S/F/S capacity that parallels dysphagia in infants and toddlers with 22q11DS. S/F/S is disrupted in *LgDel* mouse pups. We found changes in milk ingestion, transit, and distribution in *LgDel* pups. These pups have acute nasopharyngeal, as well as lung aspiration, naso-sinus/lung accumulation of milk protein accompanied by inflammation or infection in register with lymphocyte infiltration of these anomalous protein aggregates and diminished growth based on reduced rate of weight gain over the first 30 postnatal days (Karpinski et al., 2014; Yitsege et al., 2020). These disruptions parallel key features of pediatric dysphagia, including that in infants and toddlers with 22q11DS. Additional observations identify partially penetrant morphological changes in the *LgDel* mandible and midline craniofacial bones (Karpinski et al., 2014; Welby et al., 2020). The sizes, gene expression profiles, and differentiation of subsets of cranial sensory and motor neurons are disrupted in *LgDel* pups (Karpinski et al., 2014; Wang et al., 2020). Finally, altered hypoglossal motor neuron activity, including divergent dysregulation of protruder and retractor tongue muscles, is seen in *LgDel* pups (Wang et al., 2017, 2020), prefiguring craniofacial anomalies as well as disruption of tongue movement and feeding-related behaviors seen in adult *LgDel* mice (Welby et al., 2020).

It seemed possible that these disruptions of integrated craniofacial biomechanical morphogenesis, operation, and cranial sensory and motor control might be prefigured by disruption of an early developmental “program” that coordinates craniofacial and neural morphogenesis and differentiation to ensure optimal S/F/S at birth. To address this question, we first focused on potential changes in A-P hindbrain patterning that would have parallel consequences for establishing identity and signaling capacity for hindbrain cranial neural crest as well as cranial ectoderm and hindbrain neural progenitors. We reasoned that early disruption of a developmental program that coordinates face and brain development for optimal S/F/S might begin with aberrant specification of neural crest as well as neural tube progenitors of cranial/oropharyngeal skeletal elements and cranial nerve circuits (Figure 7).

We found an apparent RA-mediated “posteriorization” of anterior rhombomeres, altered expression of additional rhombomere-specific genes, and apparent increased RA signaling in posterior rhombomeres by E9.5 in the hindbrain of *LgDel* embryos (Karpinski et al., 2014; Motahari et al., 2020; Yitsege et al., 2020). This early disruption of hindbrain A-P patterning was accompanied by a 22q11 deletion-specific changes in position and initial axon outgrowth of the trigeminal nerve (CN V) in *LgDel* embryos (Karpinski et al., 2014; Maynard et al., 2020a; Motahari et al., 2020; Yitsege et al., 2020). We did not see these changes of initial CN V morphology and axon growth in *Tbx1* ^+/−^ embryos where posterior cranial nerve disruptions have been reported previously (Vitelli et al., 2002; Calmont et al., 2011, 2018). In contrast, they were enhanced in *Ranbp1* ^−/−^ embryos (Paronett et al., 2015) in which the mutant gene, *Ranbp1*, is typically expressed in premigratory neural crest and at sites of M/E induction (Maynard et al., 2002, 2003). Thus, in *Ranbp1* ^−/−^ embryos, the posterior shift of rhombomere patterning, based upon ectopic RA-regulated gene expression, is far more prominent than that in *LgDel*, as is disruption of CN V differentiation (Motahari et al., 2020).

We confirmed the RA-dependence of this hindbrain patterning change in *LgDel* and its relationship to initial cranial nerve dysmorphology *via* genetic rescue of the anomalous RA-dependent shift in patterning. We diminished RA signaling by approximately 20% (Maynard et al., 2013) using a heterozygous null allele of the rate limiting RA synthetic enzyme *Raldh2* (Zhao et al., 1996; Niederreither et al., 1999). In these compound, *LgDel*:*Raldh2* ^+/−^ embryos at E9.5, hindbrain patterning, and RA-dependent gene expression, detected by *in situ* hybridization (Figure 7) approximates the WT pattern (Figure 7), as does CN V differentiation and appropriately directed axon growth (Karpinski et al., 2014; Motahari et al., 2020). To confirm this impression based upon *in situ* hybridization, we performed qPCR for Cyp26b1 message, as well as that of three other RA-regulated genes: *Gli1*, *Rarα* and *Hoxa2*, in microdissected E9.5 hindbrains from E9.5 WT, *LgDel*, and *LgDel:Raldh2* ^+/−^ embryos. The mRNA levels for all four genes are significantly elevated above WT in the *LgDel* hindbrain and return to WT levels in the *LgDel:Raldh2* ^+/−^ hindbrain (Karpinski et al., 2014). Thus, the return of RA regulated gene expression toward WT A-P patterns and WT expression levels in *LgDel* hindbrain at E9.5 prefigures CN V differentiation in *LgDel* that is also indistinguishable from the WT by E10.5.

The RA sources that lead to altered hindbrain patterning in *LgDel* embryos remain uncertain. There is evidence that graded, as well as focal RA, signaling, activated by RA synthesized in the anterior somites as well as in the neural tube, leads to the typical RA-mediated pattern of posterior vs. anterior gene expression in the hindbrain, as well as in the differentiating cervical and lumbar spinal cord (Colbert et al., 1993; McCaffery and Drager, 1994; Maden et al., 1998; Gavalas and Krumlauf, 2000). It is uncertain whether the posteriorized pattern of gene expression in *LgDel* reflects enhanced RA production from these established sources or a shift in RA-synthetic capacity of hindbrain neural crest, migrating into the mesenchyme adjacent to anterior rhombomeres. Subsets of these neural crest mesenchymal cells produce RA (see Figure 2) once they reach the branchial arches (Bhasin et al., 2003), and they may do so ectopically to alter A-P patterning in the *LgDel* hindbrain.

These initial disruptions of hindbrain patterning, craniofacial, and cranial nerve development are accompanied by divergent differentiation of neural crest-derived cranial sensory neurons and the additional sensory and motor neurons, derived from the trigeminal placode and hindbrain neuroepithelium, respectively, with which they interact. Cell biological and lineage analysis, as well as transcriptomic comparison of WT and *LgDel* trigeminal ganglia (CNgV), indicates that the proportions of neural crest-derived and placode-derived sensory neurons in *LgDel* CNgV are altered, with placode cells and related transcripts predominating (Karpinski et al., unpublished; Maynard et al., 2020a). These changes are matched by altered mRNA transcript levels of multiple neural crest and placode-associated genes in the embryonic trigeminal ganglion (Maynard et al., 2020a), including regulators of placode (*Six1*) and neural crest-associated transcription factors (*Sox10*, *Foxd3*, *Cited4*). The proportional change of placodal vs. neural crest-derived CNgV sensory neurons reflects altered local cell-cell interactions as the ganglion coalesces that prefigure premature asymmetric neurogenic divisions by neural crest-derived CNgV progenitors (Karpinski et al., unpublished). These changes are paralleled by disrupted initial growth of CN V sensory and motor axons (Motahari et al., 2020). Aberrant axons at this early stage originate primarily in placode-derived sensory neurons or hindbrain-derived motor neurons; however, they interact extensively with anomalously patterned, transcriptionally divergent *LgDel* neural crest (Figure 7), both within CNgV and in their maxillary and mandibular targets composed largely of neural crest-derived mesenchymal cells (Motahari et al., 2020).

This early divergence of the developmental program coordinating the craniofacial periphery, cranial nerves, and hindbrain essential for optimal S/F/S prefigure dysfunction and cellular changes in cranial motor and sensory neurons in nursing *LgDel* pups. Levels of expression of genes associated with neural crest-derived nociceptive neurons in CNgV are altered in *LgDel* P8 pups (Karpinski et al., unpublished). Cranial motor neurons essential for S/F/S are also compromised in *LgDel* pups. Intrinsic excitable properties, firing rates, and effectiveness of excitatory vs. inhibitory inputs onto hypoglossal and laryngeal motor neurons are compromised in *LgDel* pups (Wang et al., 2017, 2020). These changes differ for hypoglossal motor neurons that project to protruder vs. retractor muscles of the tongue, and there are selective changes in dendritic architecture for these two target muscle-defined neuron classes (Wang et al., 2020). Additional analyses indicate similar physiological changes in laryngeal motor neurons (Caudill et al., unpublished). Finally, we found that the physiological changes in hypoglossal motor neurons are accompanied by cytological changes in distribution, morphology, and apparent neurotransmitter content of GABAergic presumed inhibitory synapses (Popratiloff et al., unpublished). Thus, multiple neuronal types, sensory neurons derived from the neural crest, as well as placode-derived sensory and hindbrain-generated motor neurons that project to oropharyngeal targets whose development relies upon the neural crest, are compromised by 22q11 deletion-dependent altered patterning of hindbrain neural crest and neural tube cells essential for morphogenetic interactions that underlie craniofacial and neuronal differentiation for optimal S/F/S.

## Many Faces (and Brains) in the Crowd

DeMyer et al. described the face’s capacity to “predict” the brain in the context of craniofacial and neurodevelopmental pathology; however, it is unlikely that this relationship serves primarily as a target for morphogenetic and behavioral pathology (LaMantia, 1999; Fish, 2016; Maynard et al., 2020b). Instead, the predictive relationship between the face and the brain may reflect adaptive flexibility that matches craniofacial specializations for sensation, as well as facial and oropharyngeal movement in individuals, as well as across vertebrate species where the cranial sensory and musculoskeletal interface for distinct environments – aquatic, terrestrial, and arboreal modes of sensory detection, breathing, food ingestion, and facial expression must be optimized for maximal adaptive advantage (Kuratani et al., 2013; Fish, 2019; York et al., 2020). This requirement for adaptive flexibility to match the face and the brain with environment and niche may be solved by deploying neural crest cells, in varying quantities with modest changes in molecular identities and genetic control networks (Depew et al., 2005; Yu, 2010; Moody and LaMantia, 2015). Once in place, similarly modest variations of neural crest/placode M/E interaction, signaling pathways, and downstream transcriptional regulation (Cotney et al., 2013; Graf et al., 2016; Dubey et al., 2018; Williams and Bohnsack, 2019; Dash and Trainor, 2020) could result in species-specific distinctions in register with demands of adaptation and selection. Such flexibility in individuals or species for neural crest as inductive ambassadors would yield substantial adaptive capacity. Accordingly, distinctions between craniofacial structures and related neural circuits in fish, frogs, birds, and mammals may reflect quantitatively modified M/E interactions that coordinate the face and the brain rather than divergent, novel mechanisms for each of these species to “put on” an adaptive face and build the neural circuits to control it effectively.

## Mirror Images: Does the Face Predict the Brain or the Brain Predict the Face?

**Figure 8:**
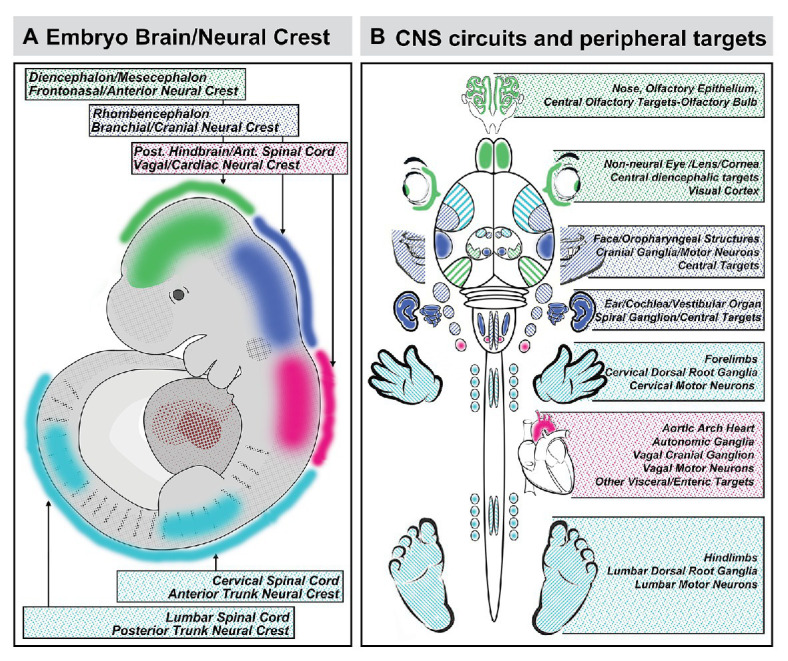
Coordination of A-P identity in the nascent central nervous system and peripheral sites of neural crest-mediated M/E induction prefigures coordinated differentiation of peripheral sensory organs, the heart and viscera, and the limbs as well as neural circuits that control each structure in the peripheral and central nervous system. **(A)** A summary of the A-P locations in the neural tube that generate neural crest and the brain regions they reflect. **(B)** The potential relationship between peripheral structures, sensory organs, and sensory ganglia induced or patterned by non-axial neural crest-mediated M/E interactions and the central neural circuits that control the function of these structures. It is unclear whether the coordination of peripheral induction *via* the neural crest in the A-P axis and corresponding regions of the neural tube has a direct influence on the regional differentiation of anterior Fb regions that process relevant information, with the exception of the OB. The locations of the relevant regions of the cerebral cortex that receive thalamic (diencephalic) inputs from relay nucleic for vision (eye), audition (ear), and somatosensation (sensory cranial ganglia) are indicated for completeness.

The sum of the evidence on coordination of craniofacial and neural development suggests that the provocative proposal of DeMyer et al. can easily be rephrased in mirror image: *the brain predicts the face*. This reflection is due primarily to the critical role played by subsets of mesenchymal neural crest cells, derived from distinct regions of the neural tube, in inducing local patterned expression of key signaling molecules and downstream effectors *via* M/E interaction to drive craniofacial and central neural circuit differentiation. Indeed, this predictive relationship between the development of neural circuits and their peripheral targets due to neural crest-mediated M/E induction is likely to constrain differentiation of the spinal cord, dorsal root ganglia, and limbs (Philippidou and Dasen, 2013), morphogenesis of the developing heart and development of its autonomic and central innervation (Vegh et al., 2016), auditory sensory differentiation and brainstem auditory circuits (Frank and Goodrich, 2018), sensory and motor circuits for cranial somatosensation (Erzurumlu et al., 2010; Kitazawa and Rijli, 2018), ocular differentiation and visual relay circuits (Petros et al., 2008; Fuhrmann, 2010), in addition to the primary olfactory pathway as well as S/F/S oropharynageal structures and circuits (Figure 8). In each instance, coordination of central neural circuit differentiation and peripheral target morphogenesis is at least constrained or at most controlled by the initial A-P identities of the neural crest cells that depart the neural tube and the neural progenitors with the same A-P identities that remain.

There is, however, an important transformation of the fundamental mechanisms for coordinated craniofacial and neural circuit development implied by the mirror image of dictum of DeMyer et al. If the brain predicts the face rather than the face predicting the brain, the fundamental pathogenic divergence in genetic or clinically diagnosed disorders that include behavioral and craniofacial disruption as key features may actually occur at the very earliest stages of *brain* development: when neural crest as well as neural stem cells in the nascent neural plate and tube begin to acquire appropriate positional identities and developmental capacities. This event precedes coalescence of the neural crest in the differentiating neural tube and its departure for M/E inductive sites (Huang and Saint-Jeannet, 2004; Sauka-Spengler and Bronner-Fraser, 2008; Stuhlmiller and Garcia-Castro, 2012). It relies upon an extensive gene and signaling network that provides a substantial set of targets for mutation, as well as environmental disruption. Thus, when the presumptive brain – neural stem cells in the neural plate and tube – is disrupted, neither the brain and the neural circuits it comprises, nor the face and the sensory specializations it will help build acquire typical, optimal states of differentiation. In this hall of mirrors, the brain does indeed predict the face before the face predicts the brain.

## Author Contributions

The sole author conceived, wrote, edited, and illustrated the manuscript.

### Conflict of Interest

The author declares that the research was conducted in the absence of any commercial or financial relationships that could be construed as a potential conflict of interest.
